# Supplementary material for: β-Catenin and SOX2 Interaction Regulate Visual Experience-Dependent Cell Homeostasis in the Developing Xenopus Thalamus
Source: Int J Mol Sci. 2023 Sep 2;24(17):13593. doi: 10.3390/ijms241713593 (PMC10488257; doi:10.3390/ijms241713593)
Supplement: Supplementary file 1 [file ijms-24-13593-s001.zip › ijms-2561920-supplementary.pdf]

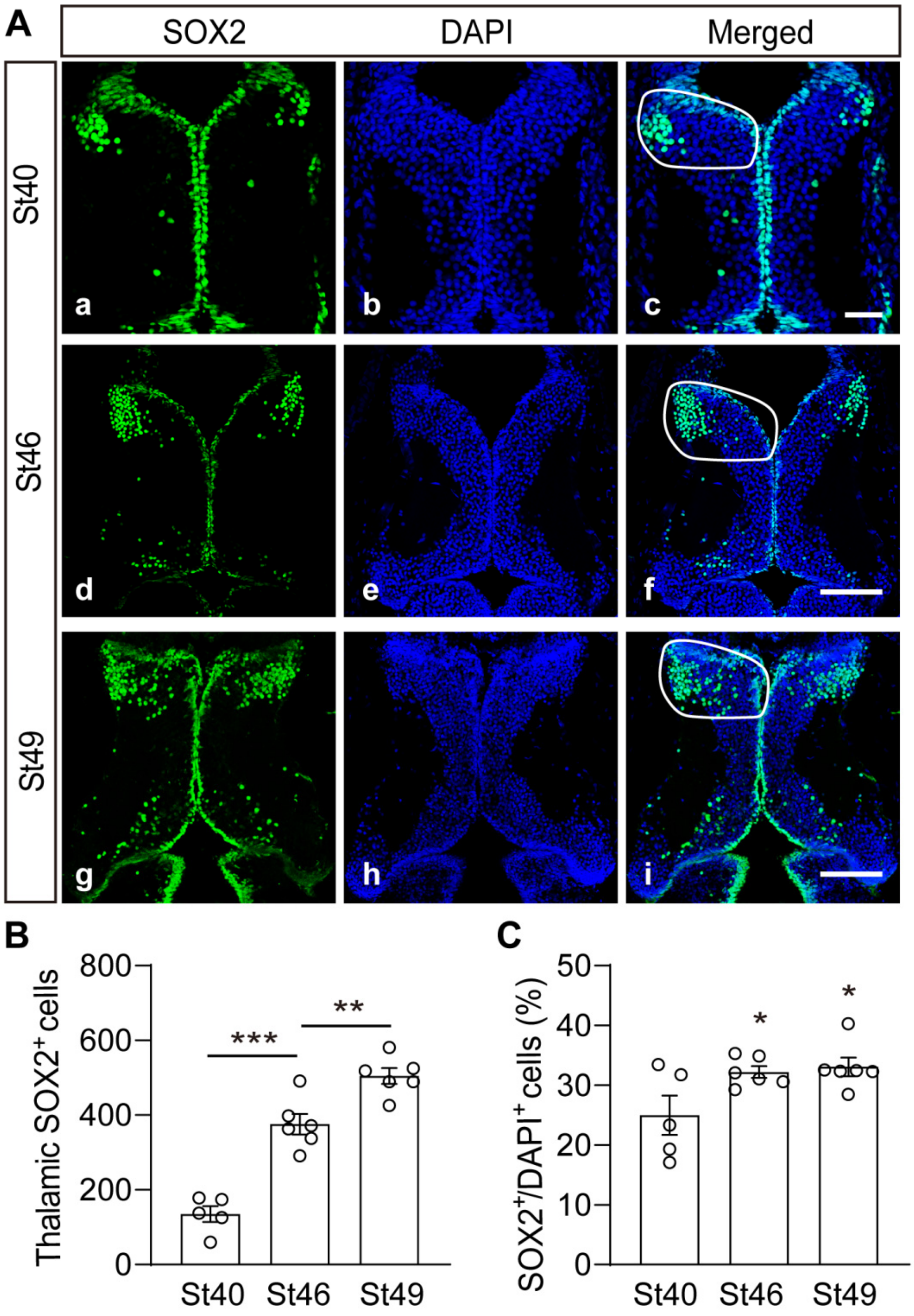

Figure S1. SOX2⁺ cells are developmentally upregulated in the thalamus.

(A-C) The brain was immunostained with an anti-SOX2 antibody at stage 40 (Aa-Ac), stage 46 (Ad-Af), and stage 49 (Ag-Ai). The white line indicates the cluster of $SOX2^+$ cells in the thalamus. Scale bar: 50 μm. (B) Summary of data showing the total number of $SOX2^+$ cells in the thalamus. (C) The summary graph shows the ratio of $SOX2^+$ cells to $DAPI^+$ cells. N = 5, 6, 6 for St40, St46 and St49; *$p < 0.05$, **$p < 0.01$, ***$p < 0.001$.

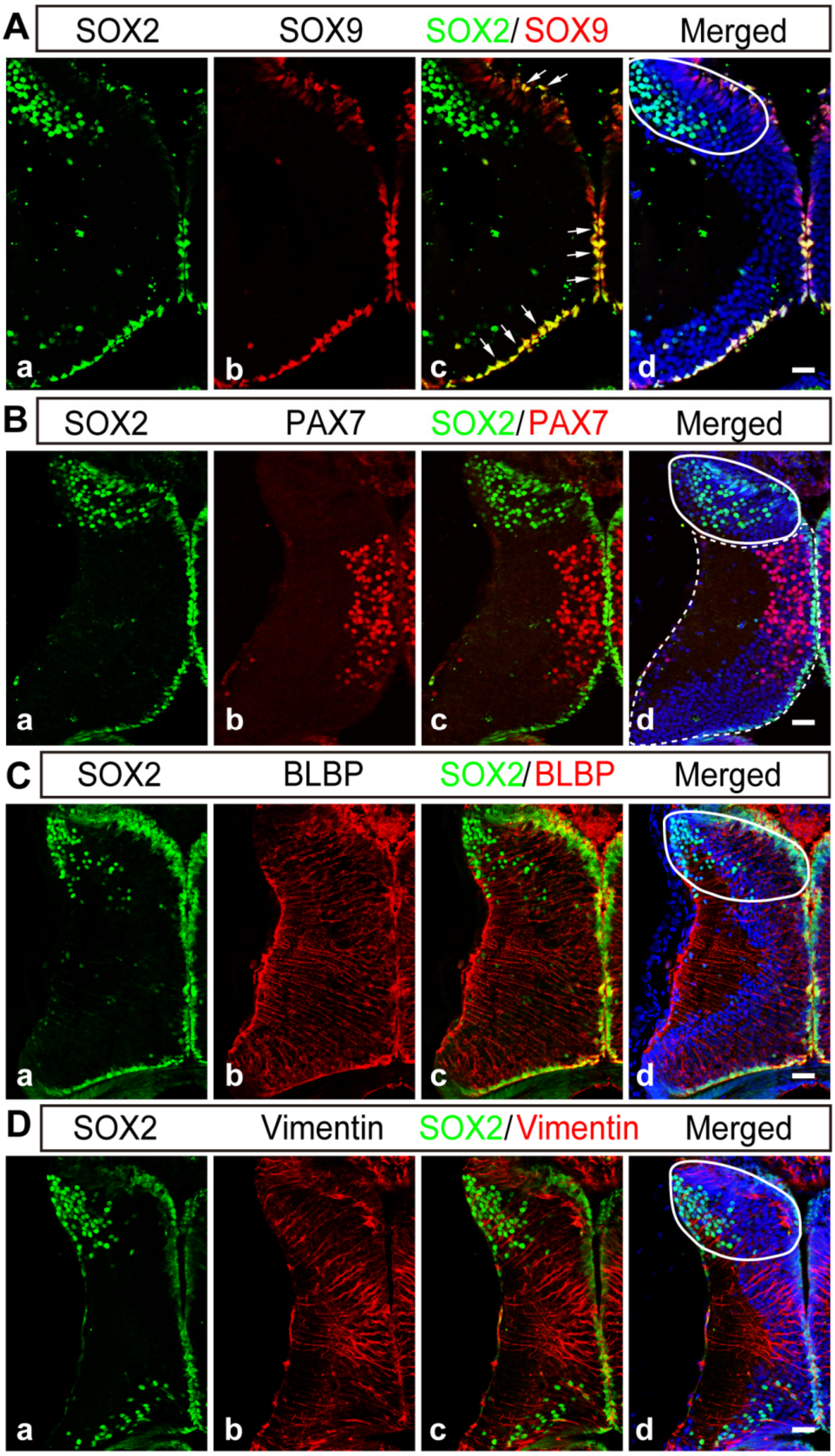

Figure S2. Colocalization of SOX2 with SOX9, PAX7, BLBP and vimentin.

(A) The brain was colabeled with anti-SOX2 (Aa) and anti-SOX9 (Ab) antibodies at stage 49. White arrows indicate the SOX2- and SOX9-labeled cells (Ac). White lines indicate the thalamus (Ad). Scale bar: 20 µm. (B) The brain was colabeled with anti-SOX2 (Ba, Bc-Bd) and anti-PAX7 (Bb, Bc-Bd) antibodies. The white line indicates the

thalamus (Ad). The white dotted line indicates the optic tectum (Ad). Scale bar: 20 μm. (C) The brain was colabeled with anti-SOX2 and anti-BLBP antibodies (Cc-Cd). Scale bar: 20 μm. (D) The brain was colabeled with anti-SOX2 and anti-vimentin antibodies (Dc-Dd). Scale bar: 20 μm.

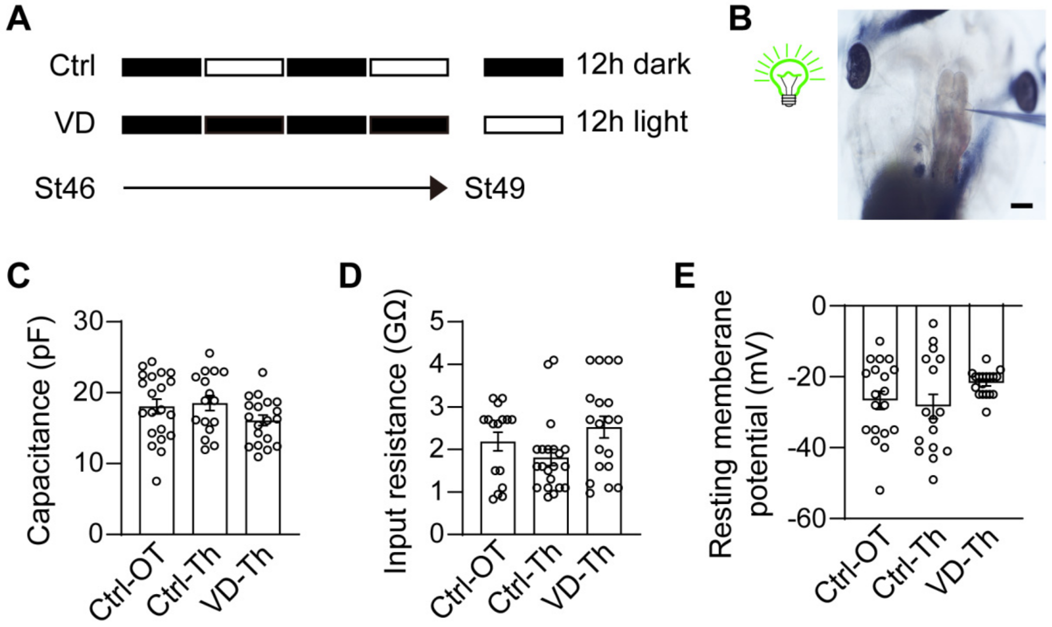

Figure S3. No changes in the electrophysiological properties of tectal and thalamic neurons.

(A) Representative illustration showing that tadpoles were placed in a light/dark box for Ctrl (12 h light/12 h dark) or visual deprivation (VD, 12 h dark/12 h dark) for 2 days. (B) Schematic diagram showing thalamic neurons were recorded in response to light stimulation. (C-E) There are no significant differences in the intrinsic cellular properties of whole-cell capacitance (C), input resistances (D) or resting membrane potentials (E) in Ctrl-OT, Ctrl-Th and VD-Th groups.

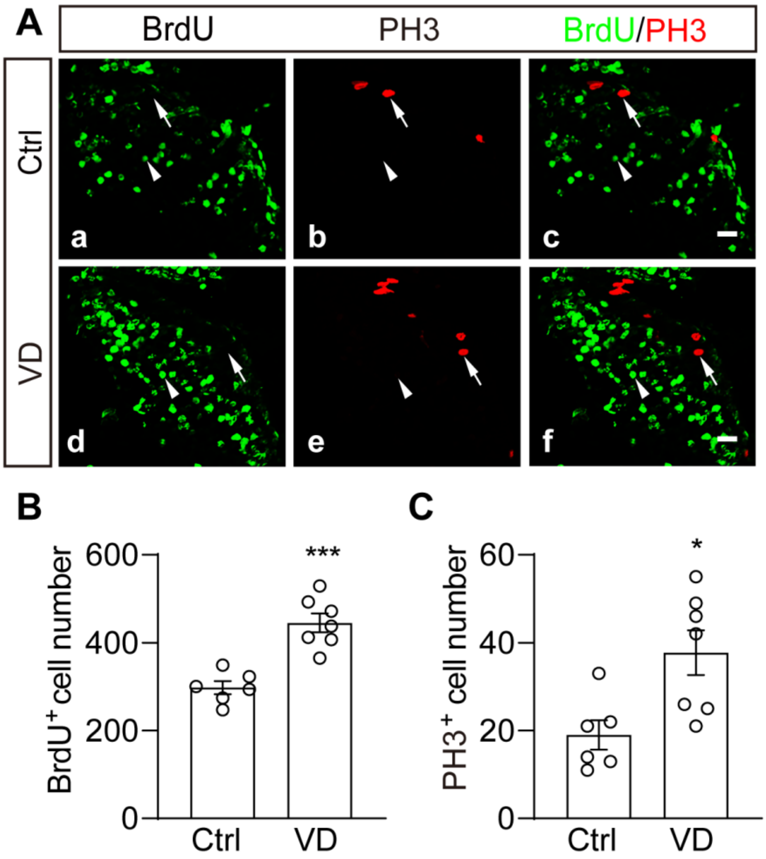

Figure S4. Visual deprivation increases PH3+ cell numbers.

(A) Representative images showing the immunolabeling of BrdU (Aa) and PH3 (Ab) in the Ctrl (Aa-Ac) and VD-treated (Ad-Af) brains. Arrows indicate the $PH3^+$ and $BrdU^-$ cells. Arrowheads indicate the $BrdU^+$ and $PH3^-$ cells in the thalamus. Scale bar: 10 μm. (B-C) The summary graph shows that VD increases $BrdU^+$ and $PH3^+$ cell numbers. N = 6, 7 for Ctrl and VD; $*p < 0.05$, $***p < 0.001$.

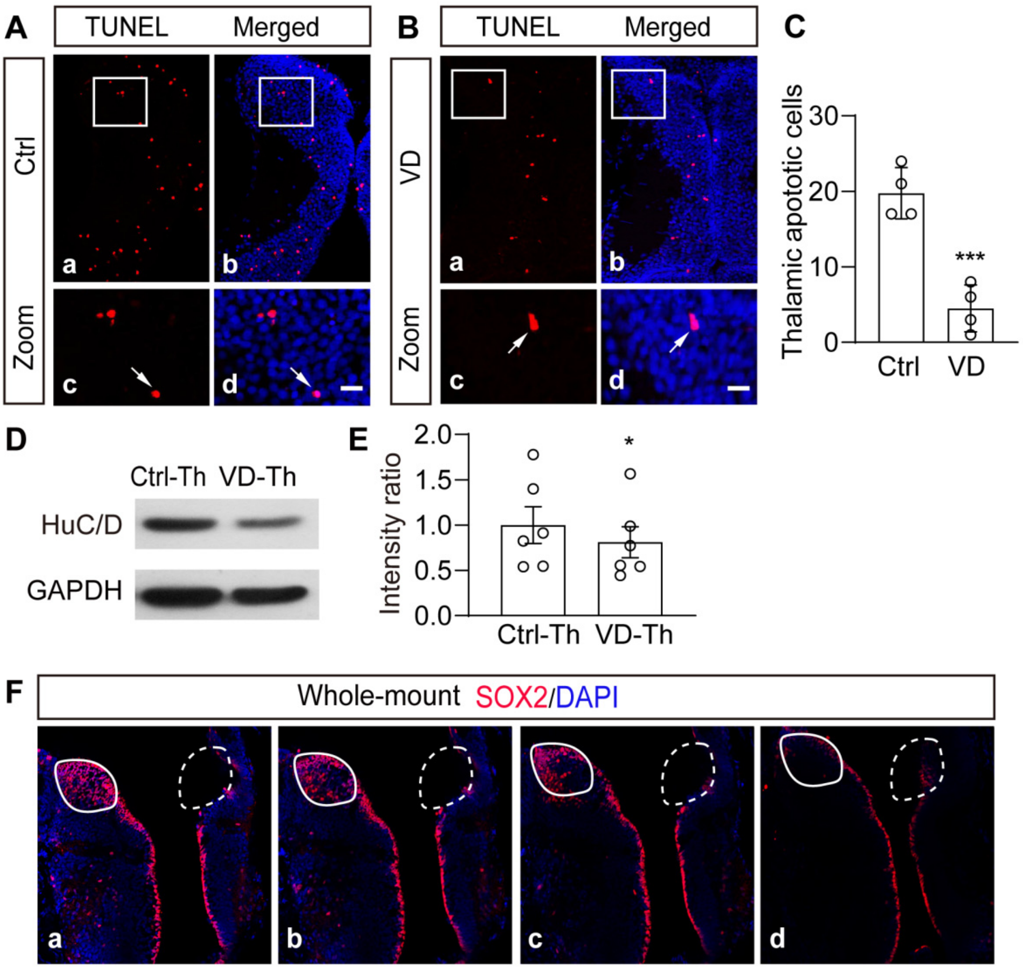

Figure S5. Visual deprivation induces decreases in neuronal differentiation.

(A-B) Representative TUNEL images showing apoptosis in the Ctrl (Aa-Ad) and VD-treated (Ba-Bd) brains. Arrows indicate the apoptotic cells in the thalamus. Zoom scale bar: 10 μm. (C) Summary of data showing that the number of apoptotic cells was significantly decreased in VD animals. N = 4, 4 for Ctrl and VD. (D-E) Western blot analysis showing that the relative intensity of HuC/D to GAPDH was significantly decreased in VD-treated tadpoles compared to Ctrl in the thalamus. N = 6. (F) The whole brain was immunostained with an anti-SOX2 antibody followed by thalamic ablation (Fa-Fd). The white dotted line indicates the ablated thalamus. The white line indicates the intact thalamus. Scale bar: 50 μm. *$p < 0.05$, ***$p < 0.001$.

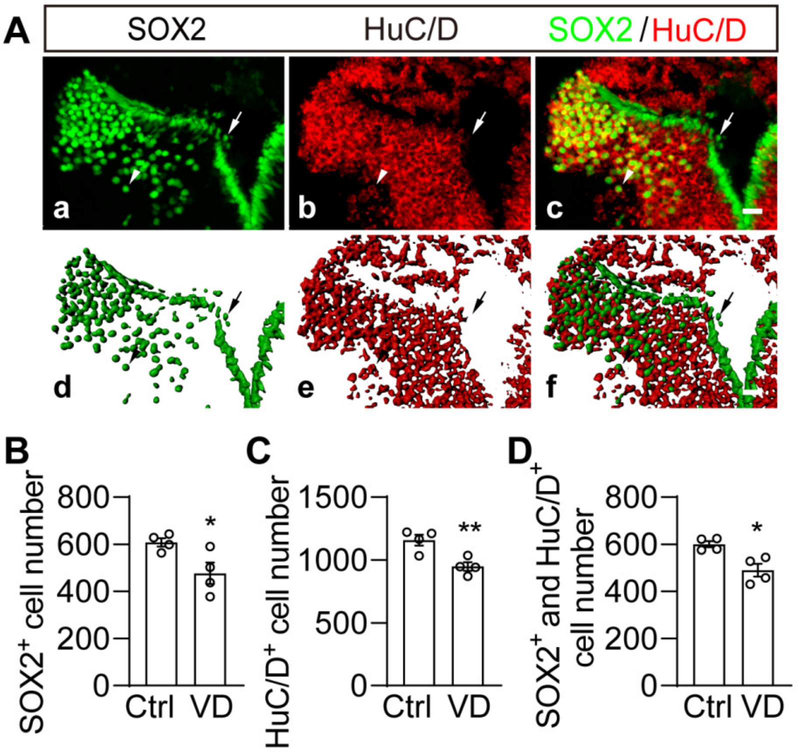

Figure S6. Cell counting for SOX2 and HuC/D by iMaris.

(A) Representative images showing the colocalization of SOX2 and HuC/D in the thalamus (Aa-Ac). The number of immunoreactive cells was counted using the surface module in iMaris (Ad-Af). Arrows indicate the $SOX2^+$ but HuC/D^- cells. Arrowheads indicate the $SOX2^+$ and HuC/D^+ cells in the thalamus. Scale bar: 10 µm. (B-D) Summary of data showing the changes in the number of $SOX2^+$ (B), HuC/D^+ (C), and $SOX2^+/HuC/D^+$ (D) cells in response to VD treatment. N = 4, 4 for Ctrl and VD; $^*p < 0.05$, $^{**}p < 0.01$.

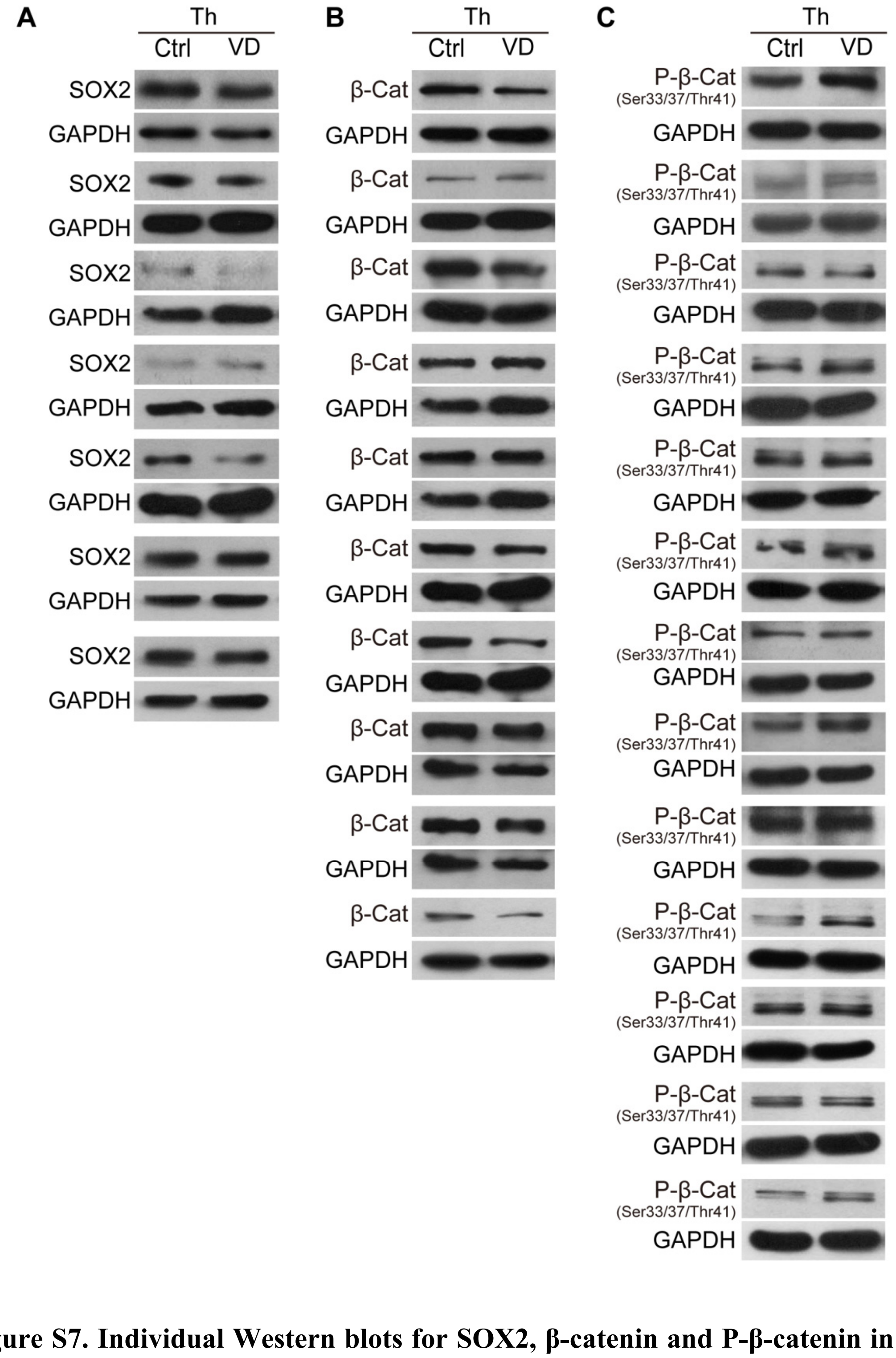

Figure S7. Individual Western blots for SOX2, β-catenin and P-β-catenin in Fig. 5E.

(A-C) Individual Western blots showing relative intensity of SOX2 (A), β-catenin (B) and P-β-catenin (C) to GAPDH in control and VD-treated tadpoles. N = 7, 10, 13 for SOX2, β-catenin and P-β-catenin.

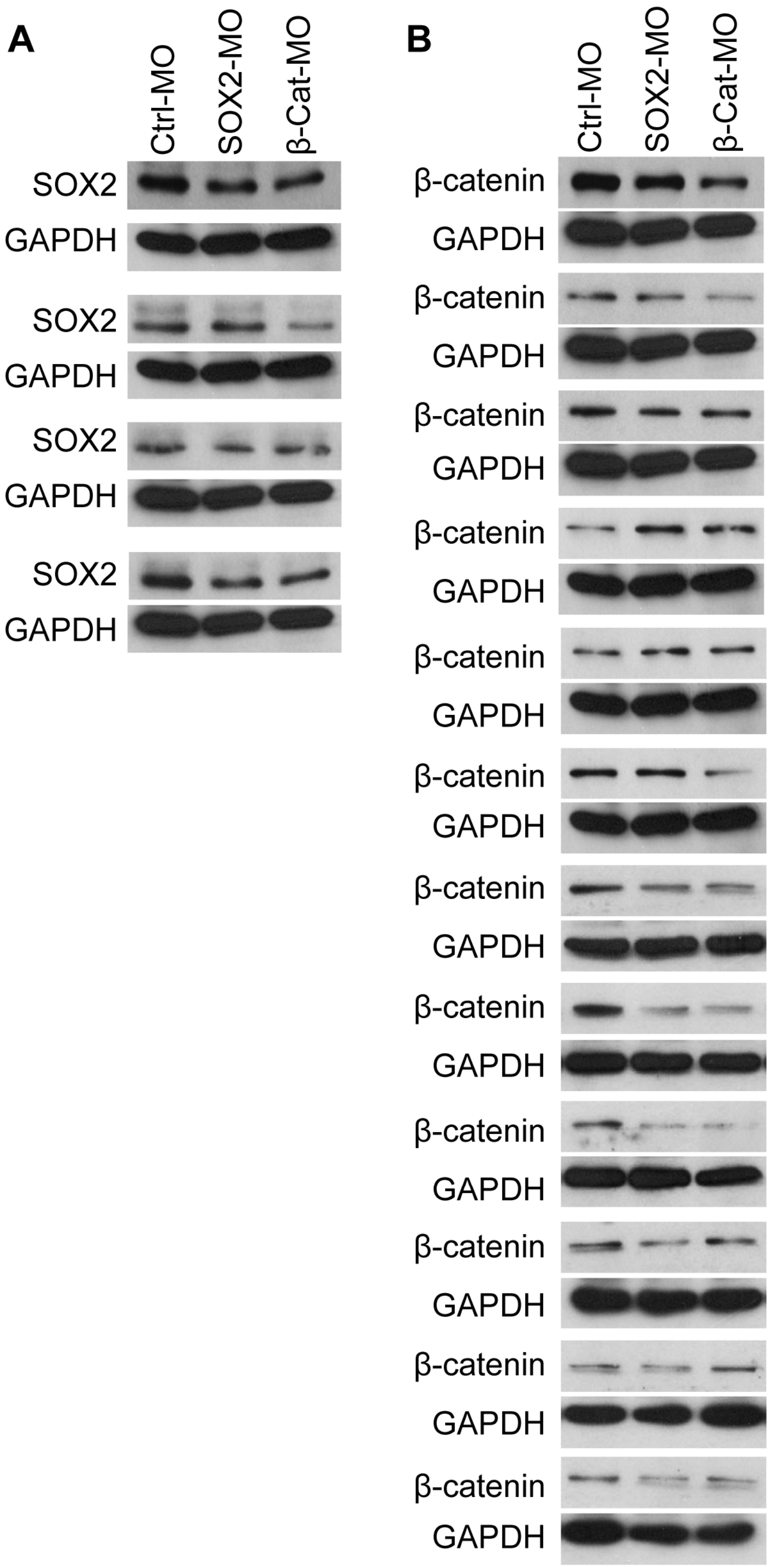

Figure S8. Individual Western blots for SOX2 and β-catenin in Fig. 6A.

(A-B) Individual Western blots showing relative intensity of SOX2 (A) and β-catenin (B) to GAPDH in Ctrl-MO, SOX2-MO and β-Cat-MO tadpoles. N = 4, 12 for SOX2, and β-catenin.

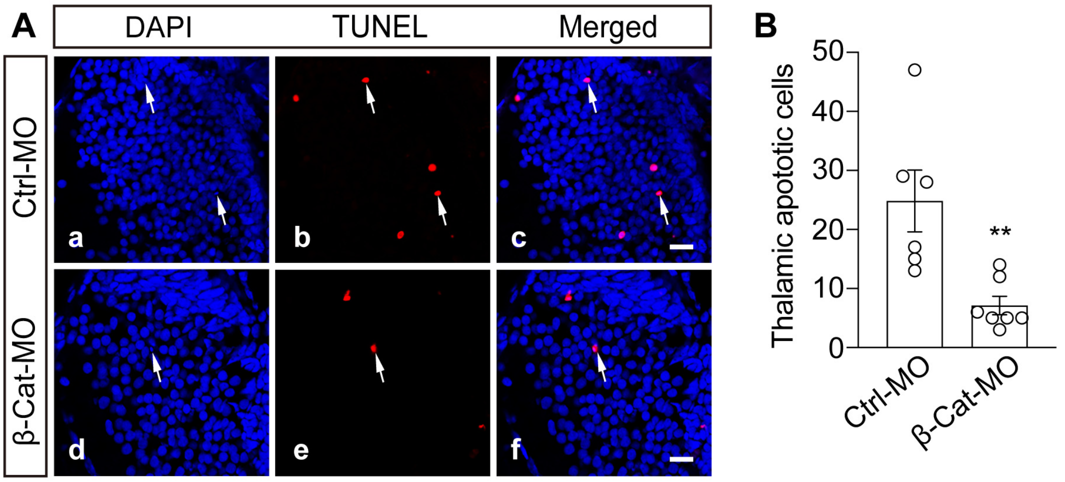

Figure S9. Knockdown of β-catenin decreases apoptotic cells.

(A) Representative TUNEL images showing apoptosis in the Ctrl-MO (Aa-Ac) and β-Cat-MO (Ad-Af) transfected thalamus. Arrows indicate the apoptotic cells in the thalamus. Scale bar: 10 μm. (B) The data show that the number of apoptotic cells was significantly decreased in the β-Cat-MO thalamus. N = 6, 7 for Ctrl and β-Cat-MO. **p < 0.01.

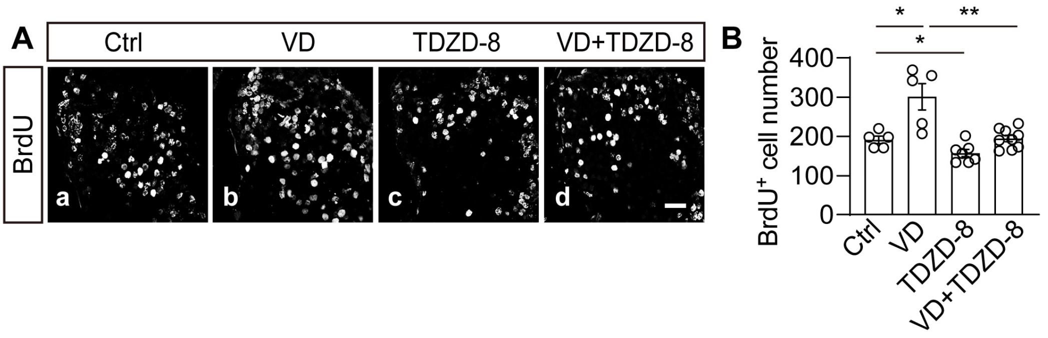

Figure S10. TDZD-8 prevented the visual deprivation-induced increase in BrdU$^+$ cells.

(A) Representative immunofluorescent images showing BrdU-labeled cells in the thalamus of Ctrl (Aa), VD (Ab), TDZD-8 (Ac), and VD+TDZD-8 (Ad) tadpoles. (B) Summary of data showing that TDZD-8 prevented the VD-induced decrease in BrdU$^+$ cells in the thalamus. N = 5, 5, 7, 9 for Ctrl, VD, TDZD-8, and VD+TDZD-8 groups. *p < 0.05, **p < 0.01.
